# Supplementary figures and images for: Dual role of the foot-and-mouth disease virus 3B1 protein in the replication complex: As protein primer and as an essential component to recruit 3Dpol to membranes
Source: PLoS Pathog. 2023 May 1;19(5):e1011373. doi: 10.1371/journal.ppat.1011373 (PMC10174528; doi:10.1371/journal.ppat.1011373)

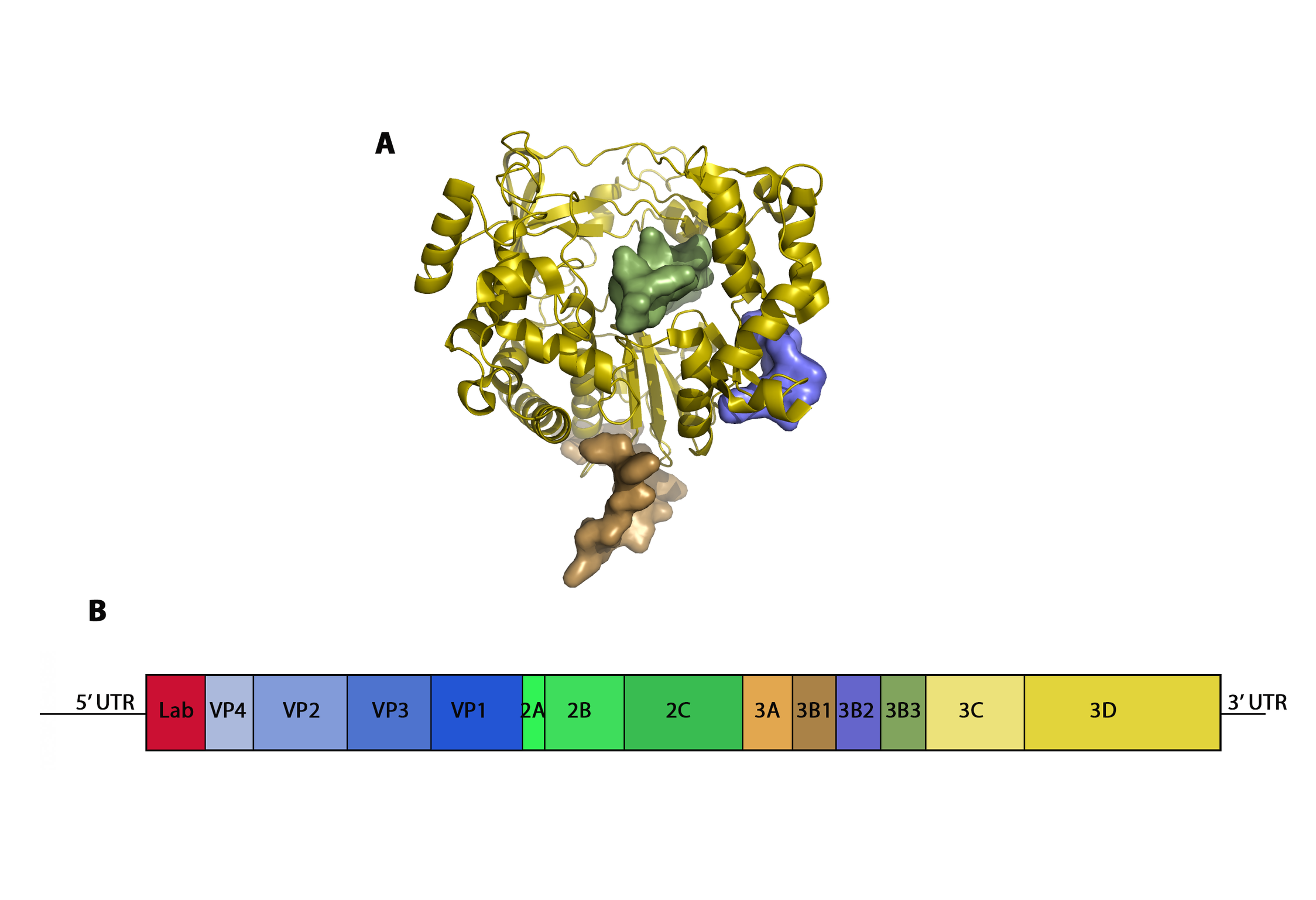

Supplement: S1 Fig — (A) FMDV 3B-3Dpol complex showing the primer peptide in green bound to active site cleft of the polymerase in yellow [20](PDB id. 2F8E), the CVB3 3B-3Dpol complex, showing 3B bound to the back side of the polymerase (in slate) [10] (PDB id. 3CDW) and the EV71 3B-3Dpol complex bound to the base of the polymerase palm (in sand) [11] (PDB id. IKA4). (B) Schematic drawing of the FMDV genome. (TIF) [file ppat.1011373.s001.tif]

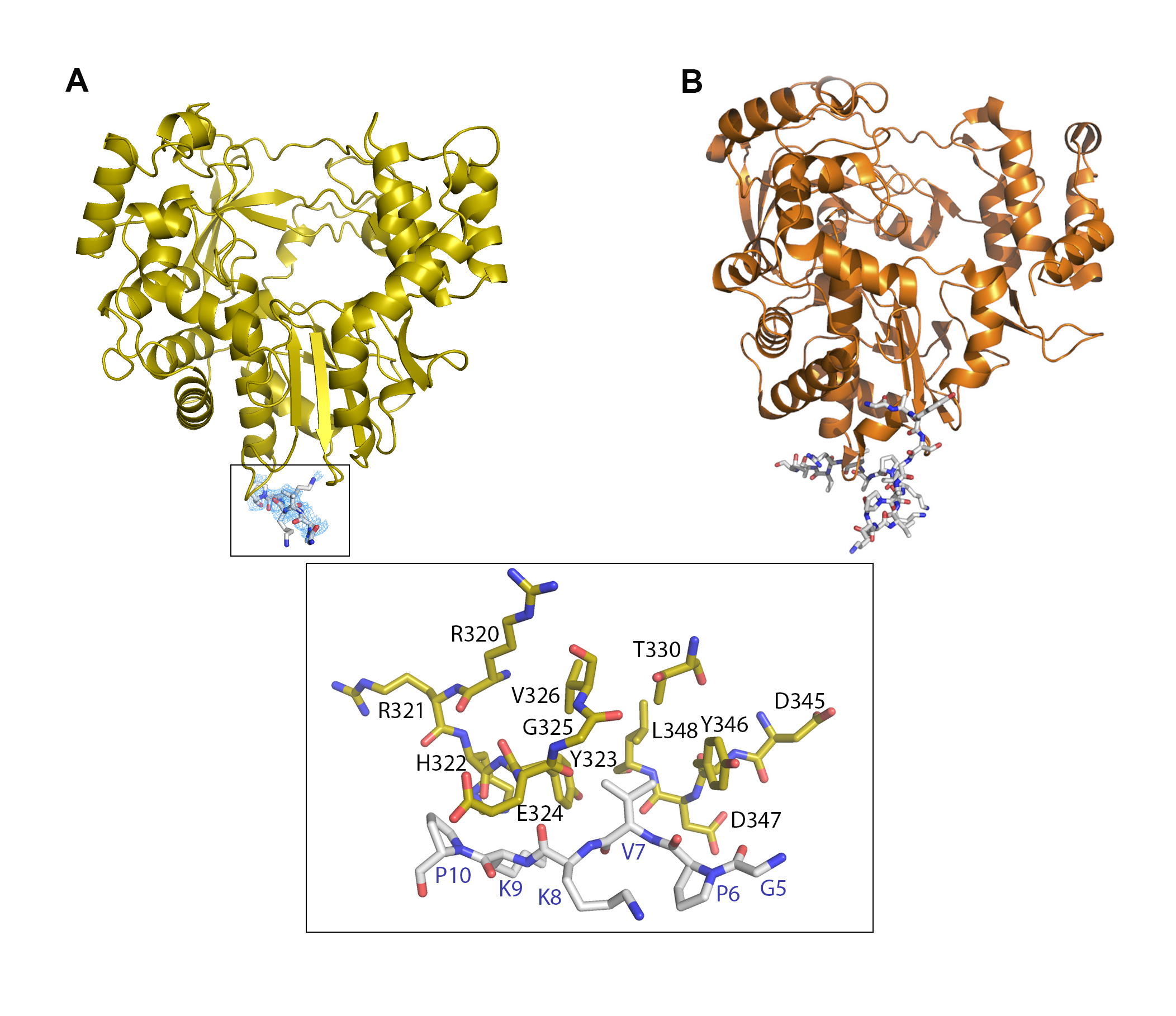

Supplement: S2 Fig — (A) Cartoon representation of the structure of the FMDV 3Dpol-3B3 complex from trigonal crystals, space group P3221. 3Dpol is shown in ribbons (yellow) and the VPg3 fragment, bound at the bottom of the palm subdomain, is shown as atom type stick (carbons in white). The corresponding 2Fo-Fc electron density map (1.0σ) is also shown around the 3B3 molecule as a light blue mesh. The proximity of a neighbouring 3Dpol molecule (Grey) in the crystal packing probably limits the 3B molecule from being arranged in an ordered way. (B) The Structure of the EV71-3B in complex with 3Dpol. 3Dpol is shown in orange ribbons and the bound VPg is depicted as atom-type sticks at the bottom of palm sub-domain [11] (PDB id 4IKA). (C) Detail of the interactions in the FMDV 3Dpol-3B3 complex. (TIF) [file ppat.1011373.s002.tif]

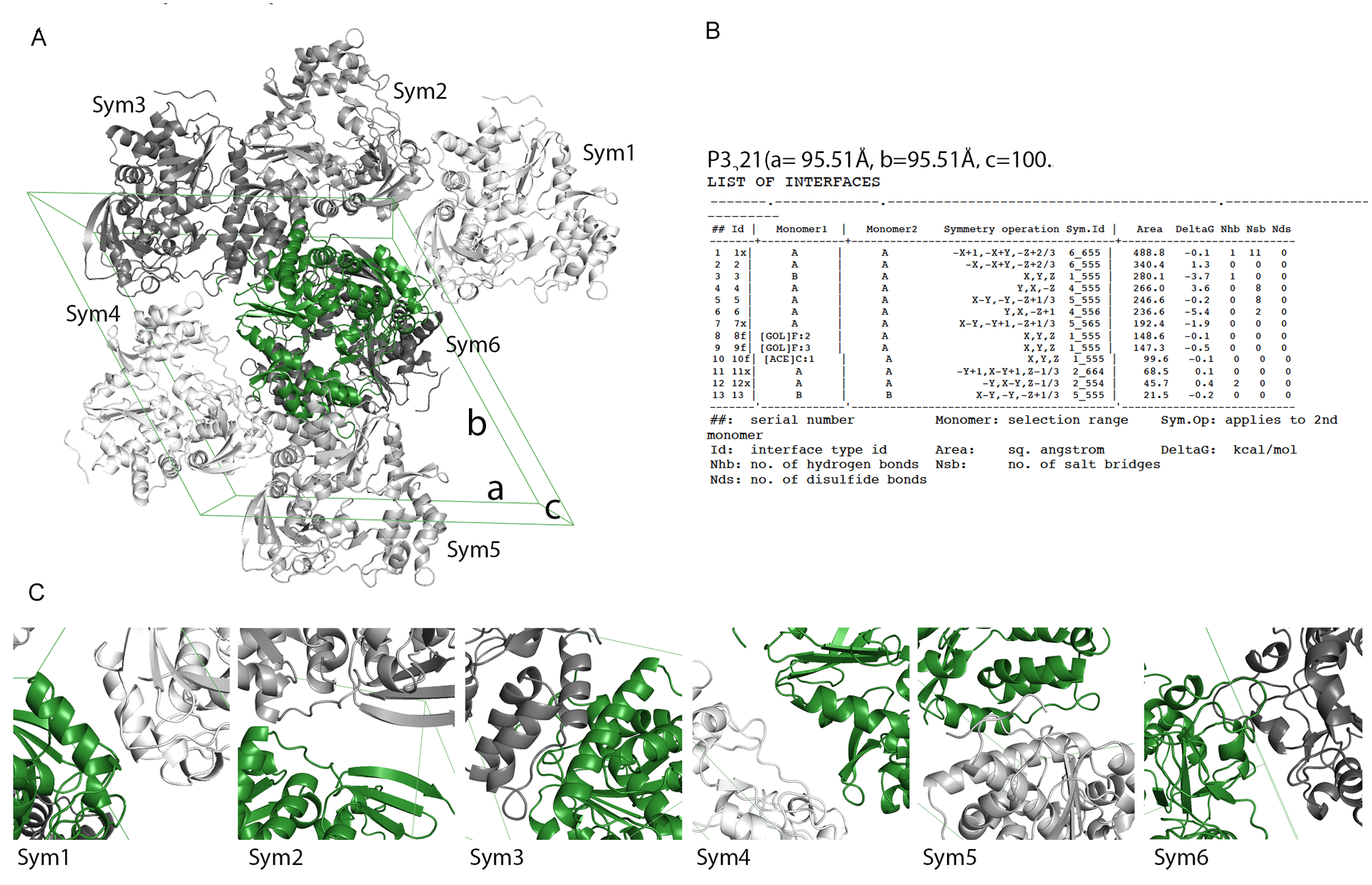

Supplement: S3 Fig — (A) 3Dpol- 3Dpol interactions in the AB plane. The reference molecule is shown in green cartoons and the contacting neighbours in grey. (B) Table showing the different contact surfaces calculated with the PISA software [41]. (C) Close up views showing the main interacting regions (TIF) [file ppat.1011373.s003.tif]

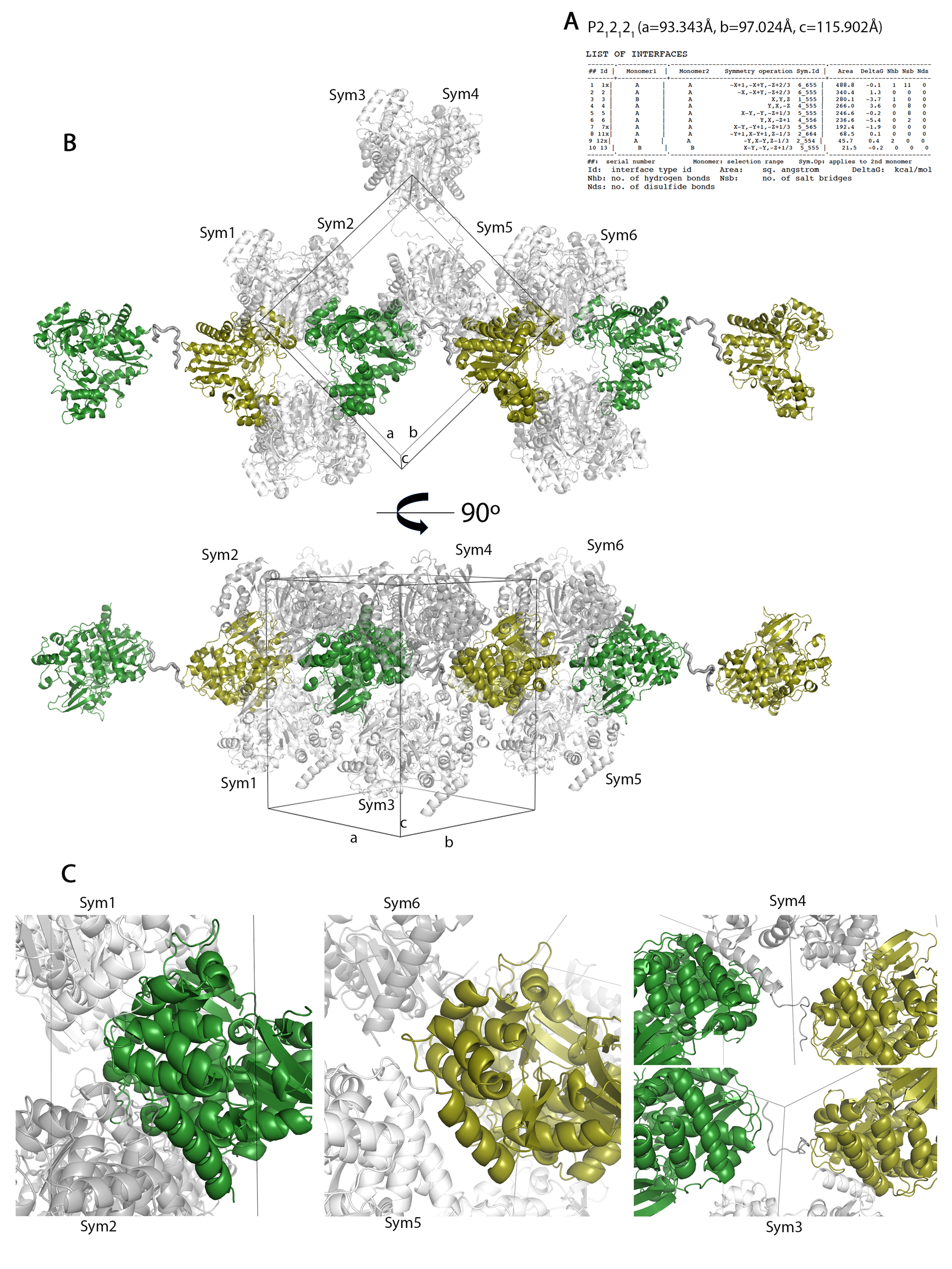

Supplement: S4 Fig — (A) Table showing the different contact surfaces calculated with PISA [41]. (B) Two different of the views of the packing contacts (related by a 90° rotation). The unit cell represented as a reference. The long 3Dpol-3B1 fibers, formed along the ab diagonal, are highlighted in green and yellow as in Fig 2, and the contacting neighbours in grey. (C) Close up views showing the main interacting regions. (TIF) [file ppat.1011373.s004.tif]

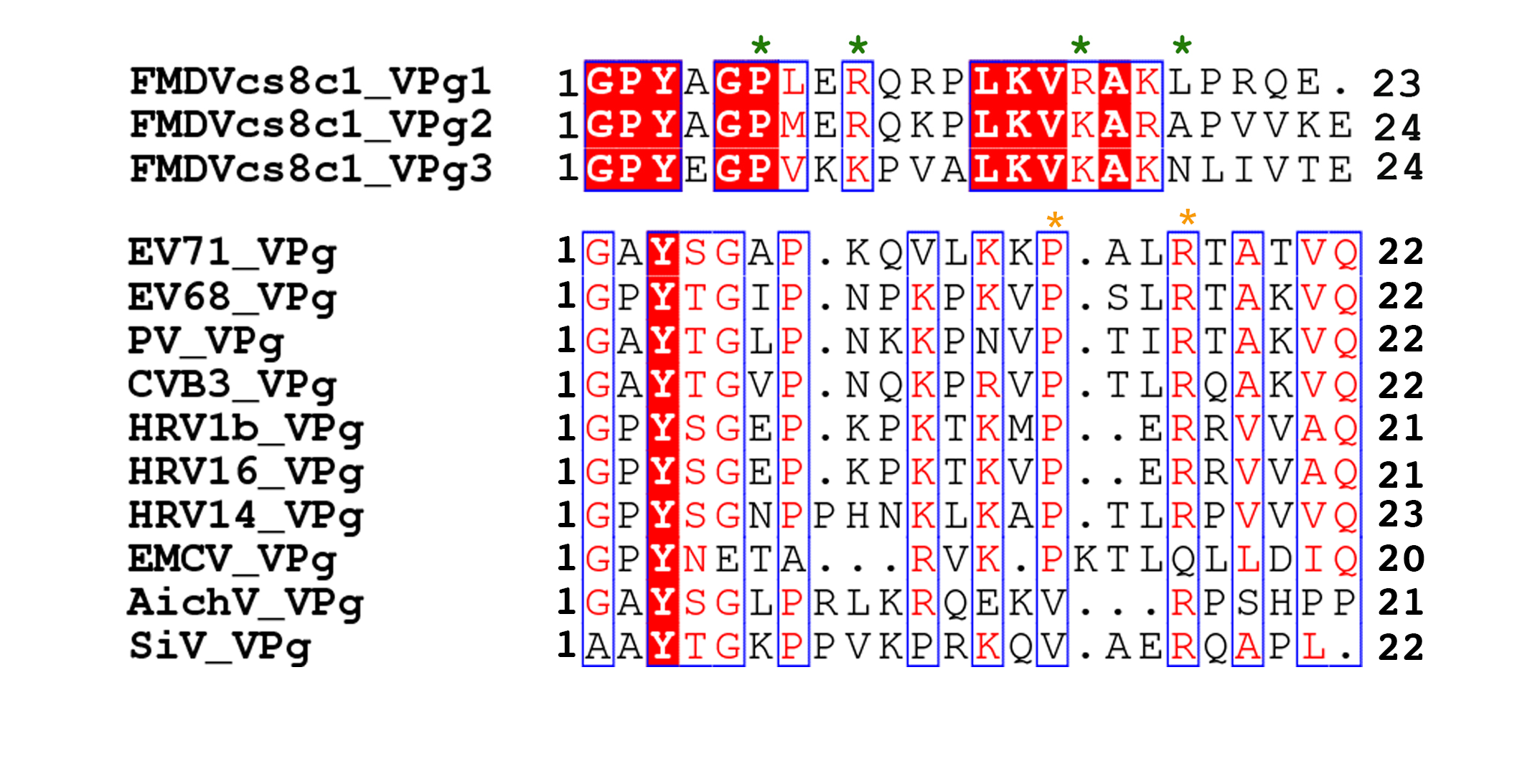

Supplement: S5 Fig — The strictly conserved residues are in red blocks and similar residues in red characters. The FMDV 3B1 residues interacting with FMDV 3Dpol are marked by green asterisks. Residues of EV71 3B previously shown to contact the bottom of the palm of EV71 3Dpol in the X-ray structure of the complex [11](PDB:4IKA) are highlighted in yellow boxes. (TIF) [file ppat.1011373.s005.tif]
