# Supplementary material for: Dual role of the foot-and-mouth disease virus 3B1 protein in the replication complex: As protein primer and as an essential component to recruit 3Dpol to membranes
Source: PLoS Pathog. 2023 May 1;19(5):e1011373. doi: 10.1371/journal.ppat.1011373 (PMC10174528; doi:10.1371/journal.ppat.1011373)
Supplement: S1 Table — (DOCX) [file ppat.1011373.s006.docx]

| **Oligonucleotide** | **Sequence (5’ to 3’)** |
| --- | --- |
| Forward to introduce 3B1 into pGEX -4T-2TEV | GTTGAAGGTGCGCGCGAAACTGCCGCGTCAGGAGTGAGCCCATATGGGAGGATCCCCGGG |
| Reverse to introduce 3B1 into pGEX -4T-2TEV | GGACGTTGCCTTTCTAGGGGTCCAGCATAGGGGCCCTGAAAATACAGGTTTTCGGTCG |
| Forward to introduce mutations P6S R9A into GST- 3B1 | CAACGTCCGTTGAAGGTGCGC |
| Reverse to introduce mutations P6S R9A into GST- 3B1 | GGCTTCTAGGCTTCCAGCATAGGGG |
| Forward to introduce mutations R16A L19S into GST- 3B1 | GCGAAAAGCCCGCGTCAGGAG |
| Reverse to introduce mutations R16A L19S into GST- 3B1 | GGCCACCTTCAACGGACGTTGCC |
| Forward to introduce mutations P6S R9A into R16A L19S GST- 3B1 | CAACGTCCGTTGAAGGTGGCC |
| Forward to introduce 3B2 into pGEX -4T-2TEV | GCTCGGGCTCCAGTAGTGAAAGAATGAGCCCATATGGGAGGATCCCCGGG |
| Reverse to introduce 3B2 into pGEX -4T-2TEV | CTTCACTTTAAGTGGTTTTTGTCTCTCCATCGGTCCGGCATACGGACCCTGAAAATACAGGTTTTCG |
| Forward to introduce 3B3 into pGEX -4T-2TEV | CAAAGAACTTAATAGTAACAGAGTGAGCCCATATGGGAGGATCCCCGGG |
| Reverse to introduce 3B3 into pGEX -4T-2TEV | CCTTCACTTTCAGTGCTACTGGCTTCTTAACCGGACCCTCGTAGGGACCCTGAAAATACAGGTTTTCG |
| Forward to introduce mutations P6S R9A into FLAG-3AB | CAGCGTCCGCTGAAGGTTCG |
| Reverse to introduce mutations P6S R9A into FLAG-3AB | GGCTTCCAGGCTACCCGCATACG |
| Forward to introduce mutations R16A L19S into FLAG-3AB | CCG CGT CAA GAA TAA GGA TCC GG |
| Reverse to introduce mutations R16A L19S into FLAG-3AB | GCTTTTCGCGGCAACCTTCAG |
| Forward to introduce mutations P6S R9A into R16A L19S FLAG-3AB | CAG CGT CCG CTG AAG GTT GC |
