# Supplementary material for: Dual role of the foot-and-mouth disease virus 3B1 protein in the replication complex: As protein primer and as an essential component to recruit 3Dpol to membranes
Source: PLoS Pathog. 2023 May 1;19(5):e1011373. doi: 10.1371/journal.ppat.1011373 (PMC10174528; doi:10.1371/journal.ppat.1011373)
Supplement: S2 Table — Summary of FMDV 3Dpol-3B complexes prepared and the number of crystals analysed that allowed obtaining X-ray diffraction data of sufficient quality to solve the structures. (DOCX) [file ppat.1011373.s007.docx]

| **3B** | **3Dpol** | **Stoichiometry**  **3B-3D^pol^** | **Other ligands** | **Diffracting Crystals** |
| --- | --- | --- | --- | --- |
| 3B1 | 3D^pol^His | 1:1 | FUTP, A10,MgCl_2_,MnCl_2_ | 3  2 |
|  |  |  | UTP, A10,MgCl2,MnCl2 |  |
|  | 3D^pol^Stop | 1:1 |  | 1 |
| 3B2 | 3D^pol^His | 1:1/1:2/1:3 | FUTP, A10,MgCl_2_,MnCl_2_ | 0  0 |
|  | 3D^pol^Stop | 1:1/1:2/1:3 | MgCl_2_ | 1 |
| 3B3 | 3D^pol^His | 1:1//1:2/1:3 | FUTP, A10,MgCl_2_,MnCl_2_  MgCl_2_ | 0  0 |
|  | 3D^pol^Stop | 1:1/1:2/1:3 | FUTP,A10,MgCl_2_,MnCl_2_  MgCl_2_ | 0  2 |
| 3B1,3B2,  3B3 | 3D^pol^His | 1:1 | MgCl_2_  MgCl_2_, UTP | 1  1 |
|  | 3D^pol^Stop | 1:1 | MgCl_2_ | 0 |
