# Supplementary material for: Dual role of the foot-and-mouth disease virus 3B1 protein in the replication complex: As protein primer and as an essential component to recruit 3Dpol to membranes
Source: PLoS Pathog. 2023 May 1;19(5):e1011373. doi: 10.1371/journal.ppat.1011373 (PMC10174528; doi:10.1371/journal.ppat.1011373)
Supplement: S3 Table — † Rwork = ∑hkl ||Fobs(hkl)|—|Fcalc(hkl)|| / ∑hkl |Fobs(hkl)|, where Fobs and Fcalc are the structure factors, deduced from measured intensities and calculated from the model, respectively. ‡ Rfree = as for Rwork but for 5% of the total reflections chosen at random and omitted from refinement. (DOCX) [file ppat.1011373.s008.docx]

| **Data collection** | 3D^pol^-3B1-UTP  (PDB id **8C1N**) | 3D^pol^Stop-3B3  (PDB id **8C2P**) |
| --- | --- | --- |
| Beamline | XALOC(ALBA) | XALOC (ALBA) |
| Resolution (Å) | 49.234- 1.7 | 46.756-1.85 |
| Space Group | P2_1_2_1_2_1_ | P3_2_21 |
| Cell dimensions |  |  |
| a, b, c (Å) | 93.343 97.024 115.902 | 95.51 95.51 100.34 |
| α, β, γ (º) | 90.0 90.0 90.0 | 90.0 90.0 120.0 |
| Rmerge | 0.060 (0.858) | 0.054 (0.671) |
| I/σI | 12.8 (1.1) | 18.9 (2.5) |
| Completeness (%) | 97.7 (87.5) | 99.3 (98.6) |
| Multiplicity | 3.6 (2.7) | 6.3 (6.4) |
| **Refinement** |  |  |
| Resolution (Å) | 49.28- 1.7 | 46.756-1.85 |
| No. reflections (total/unique) | 405084 (113370) | 275100 (43448) |
| Rwork† / Rfree‡ | 21.96/23.78 | 24.26/26.25 |
| **No. Atoms/Residues** |  |  |
| 3D^pol^ | 7474/ 943 | 3673/468 |
| VPg | 137/17 | 32/6 |
| Waters | 599/599 | 51/51 |
| **B-factors (Å^2^)** |  |  |
| All atoms | 20.71 | 46.98 |
| 3D^pol^ | 19.24 | 46.99 |
| VPg | 19.34 | 60.35 |
| **R.m.s. deviations** |  |  |
| Bond lengths (Å) | 0.0026 | 0.009 |
| Bond angles (°) | 1.166 | 1.020 |
| **Ramachandran plot** |  |  |
| Residues in preferred regions (%) | 98.5 | 97.45 |
| Residues in allowed regions (%) | 1.5 | 2.55 |

S3 Table
