# Supplementary material for: Dual role of the foot-and-mouth disease virus 3B1 protein in the replication complex: As protein primer and as an essential component to recruit 3Dpol to membranes
Source: PLoS Pathog. 2023 May 1;19(5):e1011373. doi: 10.1371/journal.ppat.1011373 (PMC10174528; doi:10.1371/journal.ppat.1011373)
Supplement: Attachment — Submitted filename: Response-to-reviewers.pdf [file ppat.1011373.s009.pdf]

Answers to Specific Comments from reviewers

Reviewer #1: none

Reviewer #2: 1) For the ELISA (Figure 4A), it would have been good to include GST alone, as a negative control as well as GST-3AB, if possible (as indicated above).

Thanks to the reviewer for the suggestion. In the revised version of the manuscript we have modified Figure 4 by adding GST alone as negative control. Unfortunately, the GST-3AB construct that contains the transmembrane helix of 3A is highly unstable in solution, forming large aggregates that do not allow a reliable study by ELISA (see SEC results below).

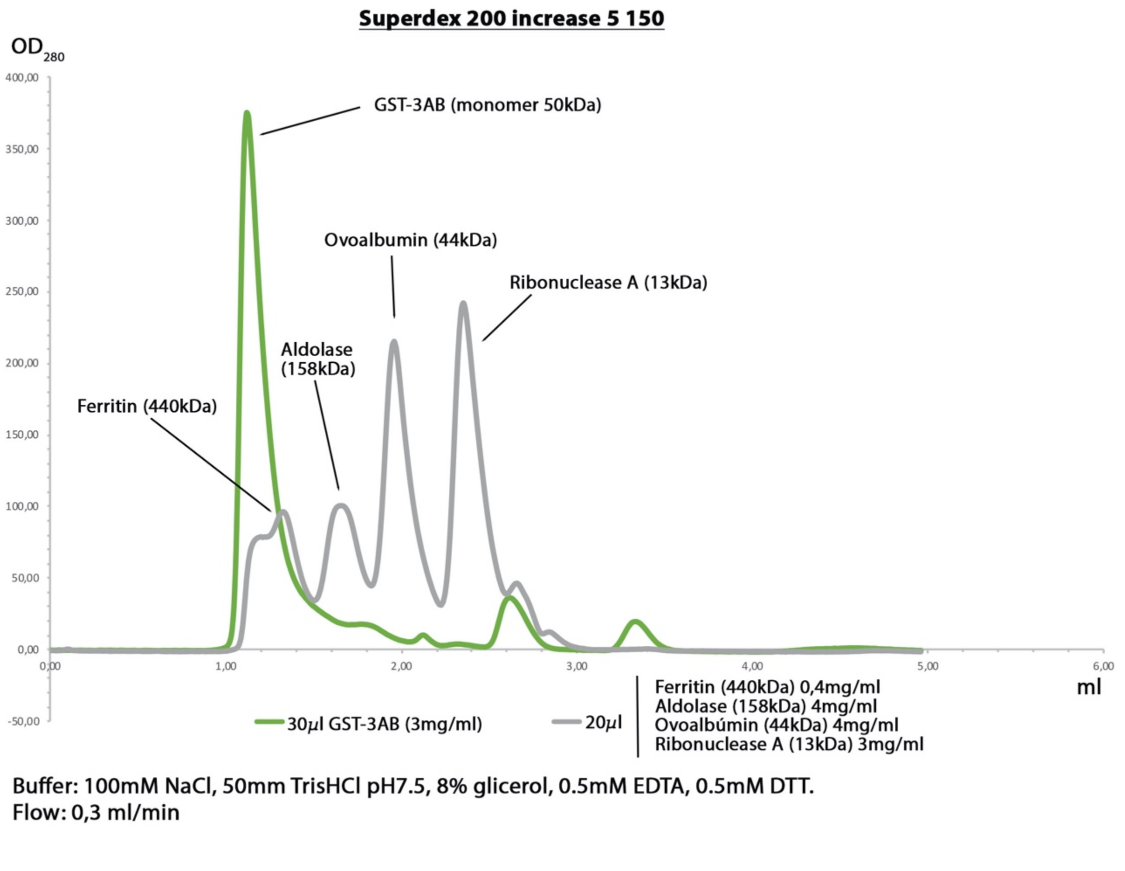

Figure SEC. Size exclusion chromatography profile of the GST-3AB construct (green profile) in buffer 50mM TrisHCl pH 7.5, 100mM NaCl 8% Glicerol 0.5 mM EDTA, 0.5mM DTT. The grey peaks show the profile of a reference protein cocktail, used as molecular weight markers.

The text, on lines 409 and 416, refers to binding affinity. However, the assays shown do not provide the binding affinity, either the text needs to be changed or the data processed to yield binding affinities (as Kd).

The text, on lines 409 and 416, has been modified in the revised version. We have replaced the word binding affinity for binding capacity. The new text reads:

409 … "*the binding capacity of 3B1 to 3D^pol^ is twice that of 3B2 and 3B3*"

416 …"*which has a lower binding capacity*"

2) The assembly of complexes on the membranes from E. coli expressing 3AB is interesting (see Fig 6). It would also have been interesting to complement the studies shown in Fig 5 with assays using the wt 3AB (in membranes) with mutant forms of the 3Dpol protein that had modifications in the different binding regions.

We thank the Reviewer for bringing up this point. Indeed, our first idea was also to study 3Dpol mutants to complement the studies summarized in Figure 5. However, a detailed analysis of the interactions showed us that 3Dpol participated in all contacts only through its main chain. See the manuscript Figure 1 and the description of the interactions provided in the text (In paragraph entitled "*3B1 binds the base of the FMDV 3D^{pol} palm, acting as a glue between two polymerase molecules in the P2$_1$2$_1$2$_1$ crystals*")

As can be seen in the Figure 1 of the manuscript and also in more detail in the figure we show below (where we have eliminated the 3D^{pol} side chains to avoid confusion), the 3B side chains P6, R9, R16 and L19 participate directly in interactions with 3D^{pol}, but there are no 3D^{pol} side chains participating in the contact interface. The fact that 3Dpol participates only with interactions mediated by the main chain makes the design of mutants virtually impossible.

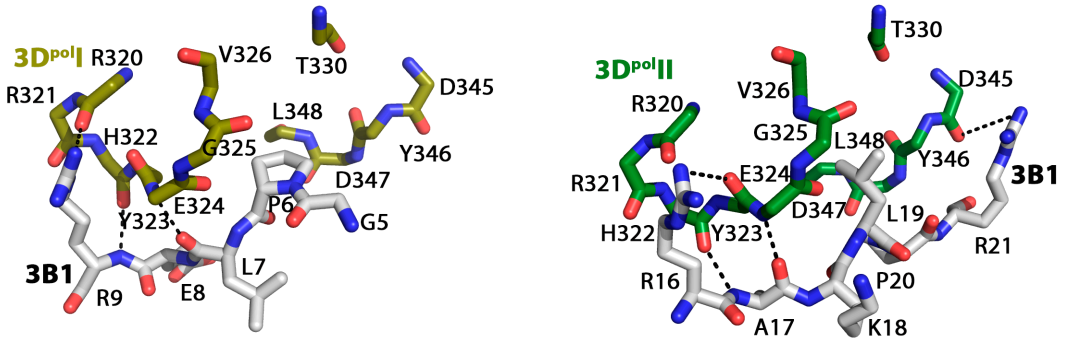

Figure Interactions. Details of the intermolecular contacts stablished between 3D^{pol} I (backbone representation as sticks, C yellow, N blue, O red), 3D^{pol} II (backbone sticks C green, N blue, O red) and the 3B1 peptide (amino acids shown as sticks in atom type colors, C white). It is important to note that the different residues of the 3D^{pol} molecules, involved in interactions with 3B1, contact only through the atoms of their main chains.

3) It would be good to test whether functional complexes are generated on the bacterial membranes, e.g. by demonstrating uridylylation in the presence of 3CD.

We appreciate this comment, unfortunately, the uridylylation experiments suggested by the referee are not ready in our laboratory, nor do we have the tools to carry out these experiments.

The main goal of the experiments carried out on bacterial membranes was to validate the new structural data, demonstrating that the binding of 3B1 to the bottom of the palm subdomain of $3D^{pol}$ is essential for the recruitment of $3D^{pol}$ to membranes. The role of the 3B1-$3D^{pol}$ interaction for polymerase recruitment to intracellular membranes was further demonstrated in cell imaging studies. Having say that, we believe that the new experiment suggested falls out of the scope of this manuscript.

Reviewer #3: Overall, the experimental work is well executed and of high quality, and there is no major need for additional experiments. The text is also well written, but there are some major inconsistencies between Figures 1-3 content and their legends that need to be remedied.

Thank you to the reviewer for pointing out this issue. Indeed, legend for panel B was missing in Figure 1 and the legends of figures 2 and 3 were exchanged in the old version of the manuscript. These errors have been corrected in the revised version.

Reviewer #1: The authors mention that it was not possible to obtain crystals of 3Dpol with 3B2. Did they perhaps try using a 3B13B2 peptide? See lines 553-4.

As shown in S2 Table, although we were able to obtain diffracting crystals of the putative $3D^{pol}$Stop-3B2 complex, we were not able to identify the position of the 3B2 peptide in the electron density maps of these crystals. Probably because the peptide was disordered.

We did not test the co-crystallization of the 3B13B2 polypeptide, what we did try was to co-crystallize the 3 combined individual peptides (3B1, 3B2 and 3B3) but the peptides did not appear ordered in the electron density maps of the diffracted crystals.

Line 81 – what does strictly conserved mean?

We intended to say that Y3 would be present in the 3B proteins of all picornaviruses.

In the revised version of the manuscript we have modified the paragraph to clarify the sentence. The new paragraph reads:

The very first step in picornavirus genome replication is the uridylylation of tyrosine 3 (Y3) in the 3B protein [16]. In this process, $3D^{pol}$ catalyzes the

successive attachment of two uridine monophosphate (UMP) molecules to the hydroxyl group of this tyrosine which is conserved among picornaviruses.

We have also replaced reference 19 with the correct review article:

Ferrer-Orta C, Agudo R, Domingo E, Verdaguer N. Structural insights into replication initiation and elongation processes by the FMDV RNA-dependent RNA polymerase. Curr Opin Struct Biol. 2009; 19(6):752-8.

Line 106-7 – have the authors checked the sequence database to ensure that this statement is correct?

Checked, see below:

FMDV is classified in 7 strains: **O** (with eleven topotypes), **A** (three topotypes), **C** (three topotypes), **Asia** (one topotype), **SAT1** (thirteen topotypes), **SAT2** (fourteen topotypes) and **SAT3** (five topotypes).

We have systematically aligned all available complete sequences of all topotypes of all strains. In all of them, we have found the three 3Bs. Here, we show a scheme with the genome region of 3B from complete genomes of different strains.

O strain

>ENA|AF308157|AF308157.1 (Shortening **O1**)
Foot-and-mouth disease virus, complete genome. Isolate name O/Yunlin/Taiwan/97. Topotype Cathay.
>ENA|AJ539141|AJ539141.1 (Shortening **O2**)
Foot-and-mouth disease virus O, strain UKG/35/2001, complete genome. Isolate name O/UKG/35/2001 Topotype Middle East-South Asia.
>ENA|AY593815|AY593815.1 (Shortening **O3**)
Foot-and-mouth disease virus O isolate o1bfs iso18, complete genome. Isolate name O_1/BFS 1860/UK/67**.** Topotype Europe -South America.
>ENA|AY593823|AY593823.1 (Shortening **O4**)
Foot-and-mouth disease virus O isolate o1manisa iso87, complete genome. Isolate name O/Manisa/TUR/69. Topotype Middle East-South Asia.

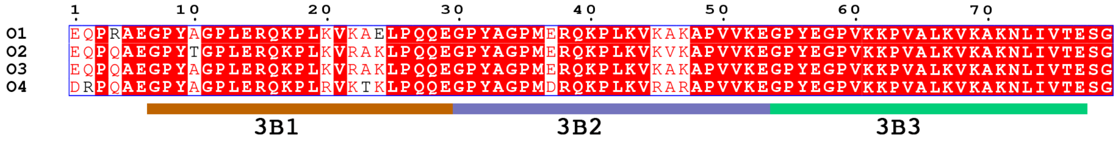

C strain

>ENA|AJ133357|AJ133357.1 (Shortening **C1**)
Foot-and-mouth disease virus (FMDV) strain C, isolate c-s8c1, genomic RNA. Isolate name C_1/Santa Pau/Spain/70 (C-S8c1). Topotype Europe- South America.
>ENA|AY593810|AY593810.1 (Shortening **C2**)
Foot-and-mouth disease virus C isolate cwald iso32, complete genome. Isolate name C/UK/149/34.

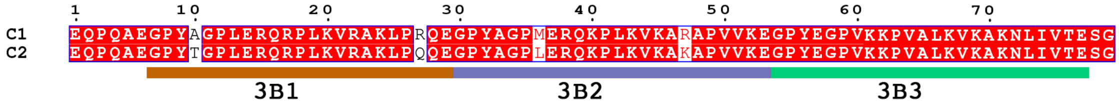

A strain

>ENA|AY593755|AY593755.1 (shortening **A1**)

Foot-and-mouth disease virus A isolate a15thailand iso43, complete genome. Isolate name A_{15}/Bangkok/TAI/60. Topotype Asia.

>ENA|AY593761|AY593761.1 (shortening **A2**)

Foot-and-mouth disease virus A isolate a21kenya iso77, complete genome. Isolate name A_{21}/Lumbwa/KEN/3/64. Topotype Africa.

>ENA|AY593763|AY593763.1 (shortening **A3**)

Foot-and-mouth disease virus A isolate a22iraq64 iso86, complete genome. Isolate name A_{22}/IRQ/24/64. Topotype Asia.

>ENA|AY593766|AY593766.1 (shortening **A4**)

Foot-and-mouth disease virus A isolate a23kenya iso8, complete genome. Isolate name A_{23}/Kitale/KEN/64 (KEN/46/65). Topotype Europe-South America.

>ENA|AY593768|AY593768.1 (shortening **A5**)

Foot-and-mouth disease virus A isolate a24cruzeiro iso71, complete genome. Isolate name A_{24}/Cruzeiro/BRA/55. Topotype Europe-South America.

>ENA|AY593780|AY593780.1 (shortening **A6**)

Foot-and-mouth disease virus A isolate a5allier iso45, complete genome. Isolate name A_5/Allier/FRA/60 Topotype Europe-South America.

>ENA|M10975|M10975.1 (shortening **A7**)

Foot & mouth disease virus A12; L, P2, and P3 polypeptide coding region. Isolate name A_{12}/119/Kent/UK/32. Topotype Europe-South America.

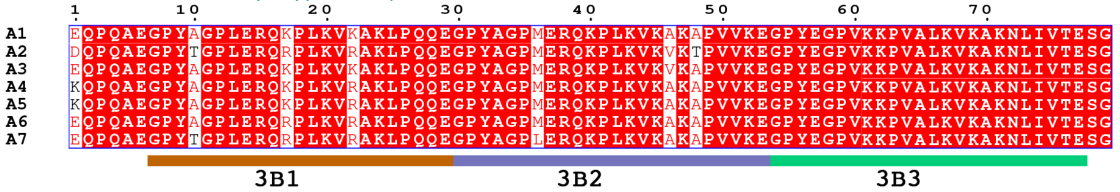

Asia-1 strain

>**ENA|AY304994|AY304994.1** (shortening **Asia1**)

Foot-and-mouth disease virus Asia 1 IND 63/72, complete genome. Isolate name Asia1/IND/63/72*. Topotype Asia.

>ENA|AY390432|AY390432.1 (shortening **Asia2**)

Foot-and-mouth disease virus Asia1 strain YNBS/58, complete genome. Isolate name Asia1/YNBS/China/58. Topotype Asia.

>ENA|AY593795|AY593795.1 (shortening **Asia3**)

Foot-and-mouth disease virus Asia 1 isolate asia1-1pak iso3, complete genome. Isolate name Asia1/PAK/1/54. Topotype Asia.

>ENA|JF739177|JF739177.1 (shortening **Asia4**)

Foot-and-mouth disease virus - type Asia 1 isolate As1/Shamir/89, complete genome. Isolate name Asia1/Shamir/ISR/89. Topotype Asia.

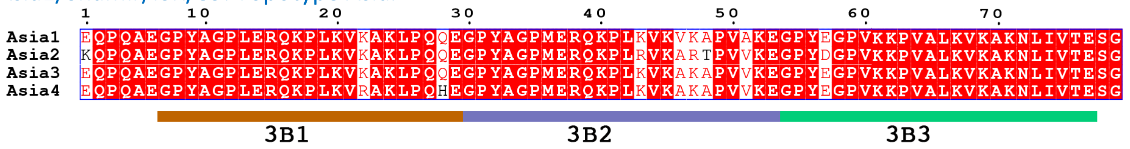

SAT1

>ENA|AY593838|AY593838.1 (shortening **SAT1.1**)

Foot-and-mouth disease virus SAT 1 isolate sat1-1bech iso30, complete genome. Isolate name SAT1/BEC/1/48. Topotype III (Western Zimbabwe).

>ENA|AY593839|AY593839.1(shortening **SAT1.2**)

Foot-and-mouth disease virus SAT 1 isolate sat1-20 iso11, complete genome. Isolate name SAT1/RV/11/37. Topotype II (Southeast Zimbabwe).

>ENA|AY593844|AY593844.1 (shortening **SAT1.3**)

Foot-and-mouth disease virus SAT 1 isolate sat1-7isrl iso12, complete genome. Isolate name SAT1/ISR/4/62.Topotype VI.

>ENA|AY593845|AY593845.1(shortening **SAT1.4**)

Foot-and-mouth disease virus SAT 1 isolate sat1bot iso47, complete genome. Isolate name SAT1/BOT/1/68. topotype III (Western Zimbabwe).

>ENA|AY593846|AY593846.1 (shortening **SAT1.5**)

Foot-and-mouth disease virus SAT 1 isolate sat1rhod iso33, complete genome. Isolate SAT1/RHO/5/66. Topotype II (Southeast Zimbabwe).

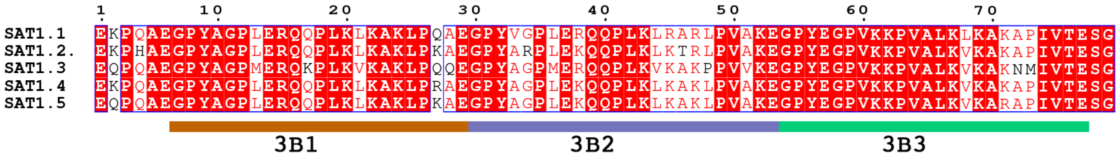

SAT-2

>ENA|AJ251473|AJ251473.1 (shortening **SAT2.1**)

Foot-and-mouth disease virus SAT2 genomic RNA for L, VP4, VP2, VP3, VP1, 2A, 2B, 2C, 3A, VPg1, VPg2, VPg3, pro coding polpolyprotein, strain KEN/3/57.Isolate SAT2/KEN/3/57. Topotype IX.

>ENA|AY593847|AY593847.1 (shortening **SAT2.2**)

Foot-and-mouth disease virus SAT 2 isolate sat2-1rhod iso26, complete genome. Isolate SAT2/RHO/1/48. Topotype III.

>ENA|AY593848|AY593848.1 (shortening **SAT2.3**)

Foot-and-mouth disease virus SAT 2 isolate sat2-2 iso25, complete genome. Isolate name SAT2/SA/106/59. Topotype I.

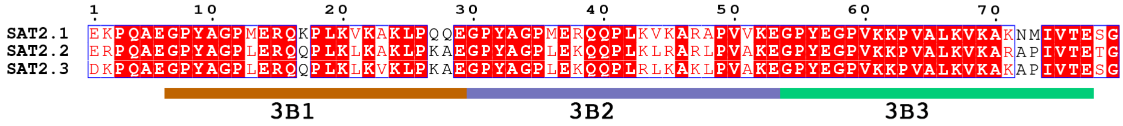

SAT3

>ENA|AY593850|AY593850.1 (shortening **SAT3.1**)

Foot-and-mouth disease virus SAT 3 isolate sat3-2sa iso27, complete genome. Isolate SAT3/SA/57/59. Topotype I (Southwest of Zimbabwe).

>ENA|AY593851|AY593851.1 (shortening **SAT3.2**)

Foot-and-mouth disease virus SAT 3 isolate sat3-3bech iso29, complete genome. Isolate name SAT3/BEC/20/61. Topotype II (Northwest Zimbabwe).

>ENA|AY593853|AY593853.1(shortening **SAT3.3**)

Foot-and-mouth disease virus SAT 3 isolate sat3-4bech iso23, complete genome. Isolate name SAT3/BEC/1/65. Topotype II (Western Zimbabwe).

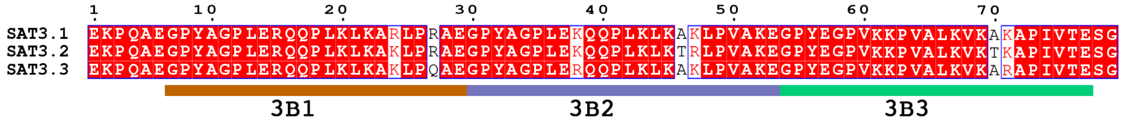

We show below the alignment of all the sequences as a summary

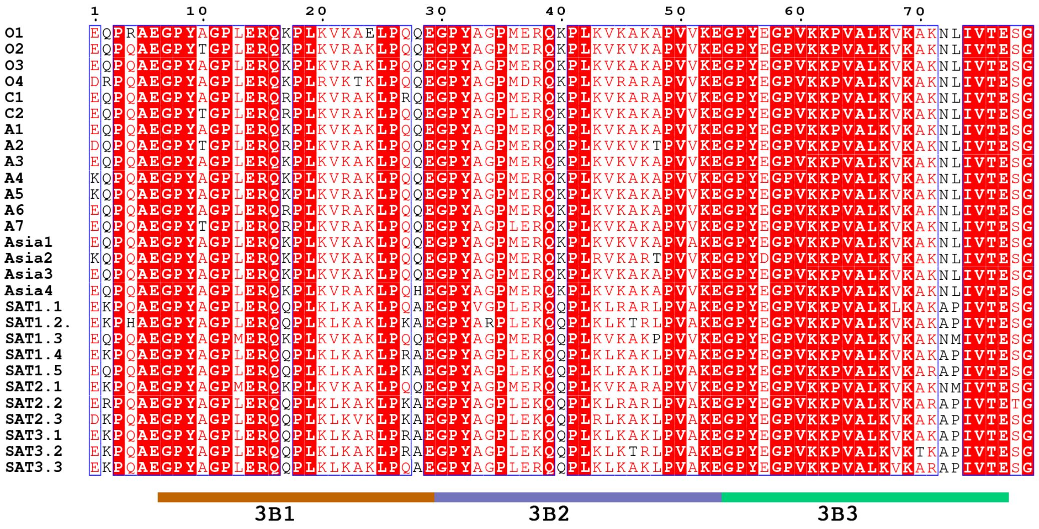

A schematic figure of the genome showing the 3Bs and 3Dpol would be a useful addition.

In the revised version of the manuscript we added a new panel (panel B) in S1 Fig, containing a schematic drawing of the FMDV genome.

It might be useful to include resolution information into the abstract.

The revised version of the manuscript includes the resolution information (1.7 Å) in the abstract

Some of the language could be improved in places, for clarity.
The following are examples and not a definitive list:

Line 99 and 523 …unlike the rest of Picornaviruses….unlike other Picornaviruses
Line 100 …all of them were found …all of these were found…
Line 325 3D polymerase…3Dpol
Line 438 On the other hand…However,
Line 501…contained…included
Line 540 How can the fibers be endless?
Line 554 should be FMDV

We thank the reviewer for these corrections. All the raised issues have been revised carefully in the text.

Reviewer #2: a) Line 433, it seems the text needs changing instead of “reinforce/stress/deeply characterize”

Revised. The new sentence reads:

To better characterize the role of 3B1-3D^{pol} interaction for polymerase recruitment….

b) There are many minor errors in the use of English that could be corrected by a native English speaker.

In the new version of the manuscript, a complete revision throughout the text has been made to correct the small errors in the use of English.

c) In the title, “essential” does not really work by itself. It could be “as an essential component to”

In the revised version of the manuscript, the title has been modified following the suggestion of the reviewer.

d) Line 199 1,5µg/ml should be 1.5µg/ml and I think on line 204 it should be 100 µl/well rather than 100ml/well. See also line 265.

Corrected in the new version of the manuscript. Old line 199 1.5µg/ml; Old line 204 100 µl/well

We also detected other minor errors (old lines 263-263) that are corrected in the revised version. The new text reads:

“HeLa S3 cells immortalized human cervical cancer cell line ECACC 93021013 were maintained at 37ºC/5% CO_2 in Dulbecco’s modified Eagle’s medium (DMEM) (Biowest) supplemented with 4.5 mg/l glucose, 584 mg/l L-glutamine, sodium pyruvate (110 mg/l; Biowest, L0104-500), 1% nonessential amino acids (MERCK)”

e) Line 215-216, it should be “site-directed mutagenesis”

Corrected in the new version of the manuscript

f) Line 232, the text refers to a western blot but it is not clear what antibody was used for this.

The revised version of the manuscript contains names and references for the antibodies used in the western blot. The new text reads:

“*The total protein concentration was normalized for the four samples by western blot, using a mouse anti-FLAG primary antibody (sigma, M2 F3165) and a Donkey anti- Mouse IgG (H+L) secondary antibody HRP (Life technologies, A16011).*”

Reviewer #3: Figure 2A is interesting in that it shows an alignment of 3Dpol fibers with intervening 3B1 structures, but is likely somewhat of a misrepresentation of all the packing interactions holding the crystal together. Are there additional polymerase-polymerase contacts in the other dimension of the P212121 and P3231 lattices that contribute to packing? Having only the 3B1 peptide just appears too flexible to yield a high resolution crystal, but perhaps not.

We thank the Reviewer for bringing up this point. Indeed, multiple polymerase-polymerase interactions contribute to the crystal packing in both space groups. In the $P2_12_12_1$ space group, each of the $3D^{pol}$ molecules of the asymmetric unit presents (in addition to the interactions shown in figure 2), other important contacts with 6 neighboring molecules in the crystal packing. This is shown in the new S4 Fig. In the revised version of the manuscript, we have added a new sentence and a supplementary figure (S4 Fig), describing the packing contacts in the $P2_12_12_1$ crystals to clarify the situation.

The new text reads:

"Beside the interactions formed between the $3D^{pol}$ dimers each individual $3D^{pol}$ molecule contacts six additional neighboring molecules, stabilizing the crystal packing (new S4 Fig)."

We have also added a supplementary figure of the packing in the $p3_221$ crystals (new S3 Fig), cited in line 338 (Clean version)

In Figure 3A, consider adding a color-matched indicator (circle?) next to the virus names for the sequences that shown in panels C-G. Or indicate virus names in panels C-G.

In the revised Fig 3, the protein names shown in panel 3A have been colored in the same colors as the molecules shown in panels B-G.
